# A Cadaveric Evaluation of Hypertrophic Obstructive Cardiomyopathy

**DOI:** 10.7759/cureus.40870

**Published:** 2023-06-23

**Authors:** Celeste M Murtha, John R Dobson, Anthony B Olinger

**Affiliations:** 1 Anatomy, College of Osteopathic Medicine, Kansas City University, Kansas City, USA; 2 Pathology and Anatomical Sciences, College of Osteopathic Medicine, Kansas City University, Kansas City, USA; 3 Pathology and Anatomical Sciences, Kansas City University, Kansas City, USA

**Keywords:** cardiac sudden death, idiopathic hypertrophic subaortic stenosis, familial hypertrophic cardiomyopathy, asymmetric septal hypertrophy, hypertrophic obstructive cardiomyopathy (hocm)

## Abstract

Hypertrophic obstructive cardiomyopathy (HOCM) describes a pathologic state in which the subaortic region of the interventricular septum undergoes significant hypertrophy and fibrosis, resulting in septal bowing into the left ventricle. The reduced left ventricular chamber size and altered cardiac function impair diastolic filling, stroke volume, and cardiac output. This case report evaluates the cardiac tissue of a 36-year-old, formalin-embalmed cadaver affected by HOCM, with the goal of providing a comprehensive overview of the gross and pathologic findings associated with the condition. This donor’s heart was found to be larger than average, weighing 510.1 g, which is 52% heavier than the predicted value of 335.6 g for a male of similar stature. The thickness of the interventricular septum, right ventricular free wall, and left ventricular free wall was comparable to other reports of HOCM. However, asymmetrical thickening of the left ventricular walls, which is characteristic of HOCM, was less prominent than expected. Histologic staining of the cadaveric tissue, with hematoxylin and eosin, trichrome, and desmin, further bolstered the diagnosis. Importantly, this also showed that histologic examination of embalmed tissue is effective and diagnostic, even 11 months after embalming. The report herein demonstrates that morphologic and histologic analysis of cadaveric cardiac tissue is sufficient to support a diagnosis of HOCM. To the researchers' knowledge, this is the first case report evaluating HOCM in a cadaver donated for medical education.

## Introduction

Hypertrophic obstructive cardiomyopathy (HOCM) is characterized by significant hypertrophy and fibrosis of the subaortic region of the interventricular septum (IVS) [1]. On gross examination, a septal wall thicker than 13 mm to 15 mm is diagnostic of HOCM [2]. In some cases, the septal wall can measure up to 50 mm [3]. The enlarged septal wall projects into the left ventricle, reducing chamber size and causing the ventricle to take on a banana-like shape [1]. Altered movement of the anterior cusp of the bicuspid valve is also a common finding, leading to aortic outflow obstruction [3]. The altered cardiac function and reduced left ventricular chamber size impair compliance, diastolic filling, stroke volume, and cardiac output.

This condition was first described in 1868 by Dr. Alfred Vulpian, who termed it “idiopathic hypertrophic subaortic stenosis” (IHSS). For the next century, physicians continued to describe rare reports of similar findings on postmortem examination [4]. Idiopathic hypertrophic subaortic stenosis was recognized as a distinct disease in 1961 and renamed hypertrophic obstructive cardiomyopathy in the late 20th century [4]. Hypertrophic obstructive cardiomyopathy affects 0.2% of the population with an annual mortality rate of 0.05% [3]. The condition is most often attributed to autosomal dominant mutations in sarcomere proteins responsible for myocardial contraction [2]. Mutations in β-myosin heavy chain, myosin binding protein C, and troponin T account for 80% of genetically attributed cases [2]. These gain-of-function mutations cause myocyte hypercontractility, inducing cardiomyocyte hypertrophy and asymmetrical thickening of the left ventricular walls. However, genetic causes account for only 30% of diagnosed cases [3]. A recent Italian study analyzing 1,200 patients with suspected HOCM reported that genetic screening yielded pathogenic variants in 47%, suggesting that unknown epigenetic factors are also at play [5]. Even within families, the disease can manifest heterogeneously, suggesting that sarcomere mutations are not the only factors dictating the disease phenotype [3].

Diagnosis relies on the detection of left ventricular hypertrophy that cannot be attributed to other cardiac or systemic pathology. Echocardiogram, electrocardiogram, and cardiovascular MRI are modalities useful in the diagnostic algorithm [3]. On echocardiogram, the presence of a hypertrophied and nondilated left ventricle with septal thickness ≥15 mm is suggestive of HOCM [2,6]. Histologic analysis of the cardiac tissue, taken during endomyocardial biopsy or on postmortem evaluation, can also support the diagnosis and rule out other infiltrative etiologies (i.e., amyloidosis, sarcoidosis, hemochromatosis). Characteristic histologic features include myocyte hypertrophy (transverse myocyte diameter >40 µm), myofiber disarray, nuclear enlargement and hyperchromasia, and interstitial fibrosis [1,5].

Physical examination often reveals a systolic ejection murmur due to obstruction of the left ventricular outflow tract. Symptoms are variable and present in approximately 50% of patients [7]. Patients may experience reduced exercise capacity, exertional chest pain, dyspnea, and syncope. Sequelae include atrial fibrillation, ventricular fibrillation, stroke, infectious endocarditis, myocardial ischemia, and congestive heart failure [1]. The most serious complication is sudden death due to arrhythmia [8]. In fact, 36% of cases of sudden death among competitive athletes are attributed to HOCM [3]. Fortunately, HOCM can be managed with pharmacologic and/or surgical intervention, allowing many patients to enjoy a normal lifespan [3].

This report explores the morphologic and histologic changes secondary to HOCM in a 36-year-old male cadaver. A comparison of heart size (i.e., weight, wall thickness, etc.) among the affected donor and the average male will demonstrate the gross effects that this condition can elicit and will highlight the phenotypic variability that exists within the condition. Furthermore, this report shows that histologic examination of embalmed tissue is effective and diagnostic, even 11 months after embalming.

## Case presentation

This report details a case of HOCM in a 36-year-old male cadaver with a height of 170.2 cm, weight of 65.8 kg, and BMI of 22.7 (before embalming). This patient’s cause of death was reported as hypoxic respiratory failure and left middle cerebral artery stroke. Limited medical records revealed a history of spontaneous intestinal perforation, knee surgery, hypertension, replacement of bicuspid and aortic valves, and surgical placement of a pacemaker and implantable cardioverter-defibrillator (ICD). While it cannot be said with absolute certainty due to the lack of comprehensive medical records, it is likely that this patient's cardiac procedures (valve replacement, ICD/pacemaker placement) were secondary to his HOCM diagnosis. 

Anatomic evaluation

**Figure 1:**
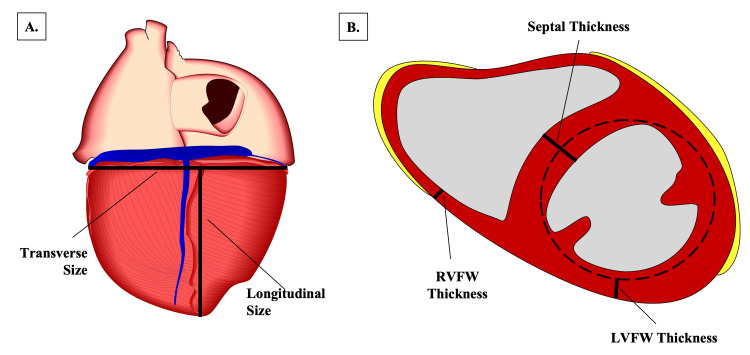
Cardiac tissue measurements A: Depiction of transverse and longitudinal measurements; B: Depiction of RVFW, LVFW, and septal thickness measurements Diagram created by Dr. Anthony Olinger, informed by research conducted by Basso et al. [9]. RVFW: Right ventricular free wall, LVFW: Left ventricular free wall

The donor’s heart was removed from the mediastinum, cleaned of fat and fascia, and all postmortem clots were cleared. On initial examination of the chest cavity, a pacemaker/ICD was observed. Further exploration of the cardiac tissue also revealed two mechanical bi-leaflet valves, replacing the native bicuspid and aortic valves. The aorta and pulmonary trunks were transected 1 cm above their origin. The cardiac tissue was then weighed using a digital tabletop scale (Scout Balance Scale, OHAUS Instruments Co. Ltd., Parsippany, NJ, USA), and a ruler was utilized to measure heart dimensions, per the methodology described by Basso et al. [9]. The transverse size was measured at the broadest part of the transverse diameter of the heart (Figure 1A) [9]. The longitudinal size was measured from the apex of the heart to the base along the midline (Figure 1A) [9]. The heart was subsequently cut in the transverse plane to produce several 1 cm tissue slices that exposed both the right and left ventricular cavities (Figure 1B). At the mid-ventricular level, the thickness of the left ventricular free wall (LVFW), right ventricular free wall (RVFW), and IVS was measured [9]. Papillary muscles and trabeculae carneae were excluded when measuring wall thickness [9]. Lastly, tissue samples were taken from the LVFW, RVFW, and IVS at the mid-ventricular level. These samples were placed in cassettes and bathed in 10% formalin before processing and staining by Mizzou OneHealth Biorepository (Columbia, MO). The IVS was stained with hematoxylin and eosin (H&E), Masson’s trichrome, and desmin. The RVFW and LVFW were stained with H&E only. Control stains were performed via the same methodology, using cardiac tissue from a cadaver without known cardiac conditions. Slides were viewed under a microscope (Leica DM500, Leica Microsystems, Wetzlar, Germany); pictures and cell measurements were taken using the View4K High-Definition Microscope Camera (Microscope Central, Feasterville, PA, USA) and the MIImageView software.

Findings

**Figure 2:**
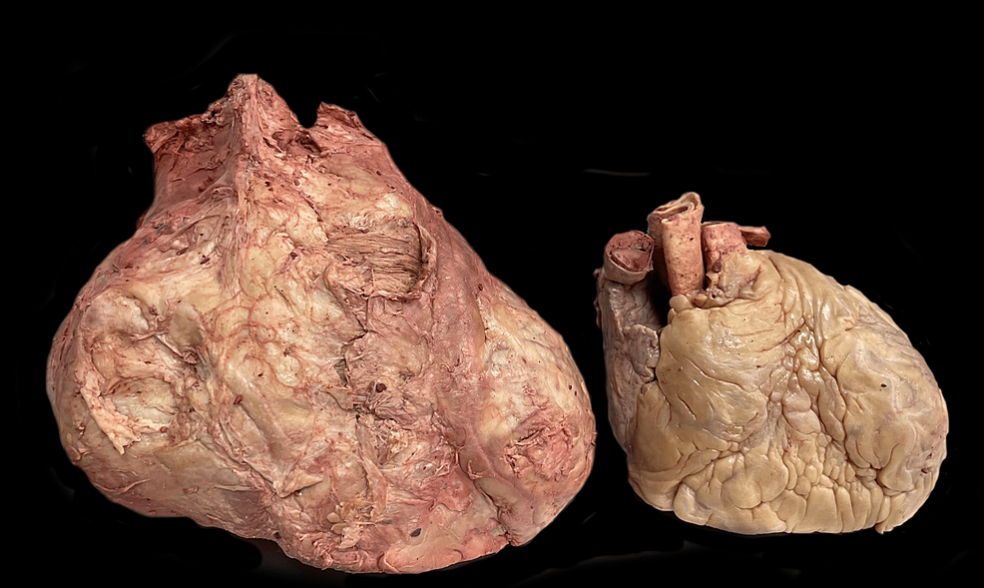
Side-by-side comparison of the heart affected by HOCM (left) and an unaffected heart (right) HOCM: Hypertrophic obstructive cardiomyopathy

**Figure 3:**
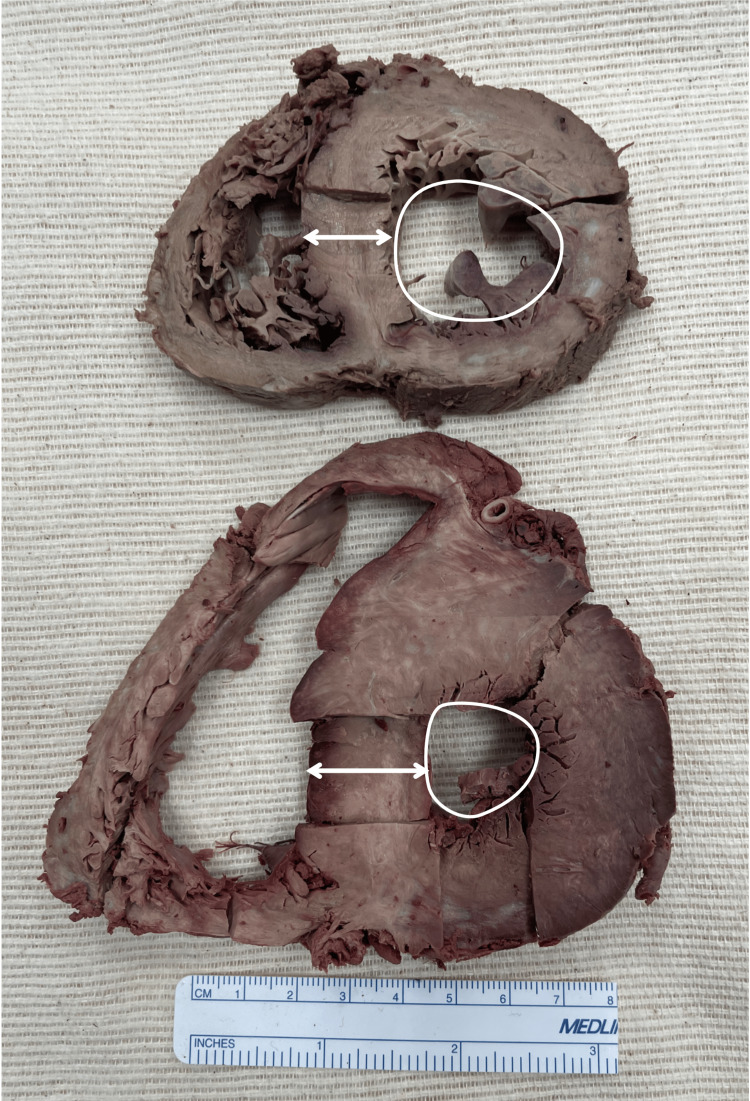
Comparison of transverse sections of the unaffected heart (top) and HOCM-affected heart (bottom) demonstrates a gross difference in general heart size, septal thickness (double-ended arrow), and left ventricular chamber size (circle) HOCM: Hypertrophic obstructive cardiomyopathy

This donor’s heart was grossly larger than the average heart, weighing 510.1 g (Figure 2). Longitudinal and transverse sizes of the heart were 9.7 cm and 12 cm, respectively. The IVS, RVFW, and LVFW thicknesses were 21 mm, 4 mm, and 16 mm, respectively (Table 1). Figure 3 compares transverse sections of unaffected and affected cardiac tissue, showing a clear difference in septal thickness, left ventricular wall thickness, and left ventricular chamber size.

**Table 1:** Summary of size metrics for cadaveric heart affected by HOCM HOCM: Hypertrophic obstructive cardiomyopathy, IVS: Interventricular septum, RVFW: Right ventricular free wall, LVFW: Left ventricular free wall

Parameters	Donor heart
Weight (g)	510.1
Longitudinal size (cm)	9.7
Transverse size (cm)	12.0
IVS thickness (mm)	21.0
RVFW thickness (mm)	4.0
LVFW thickness (mm)	16.0

**Figure 4:**
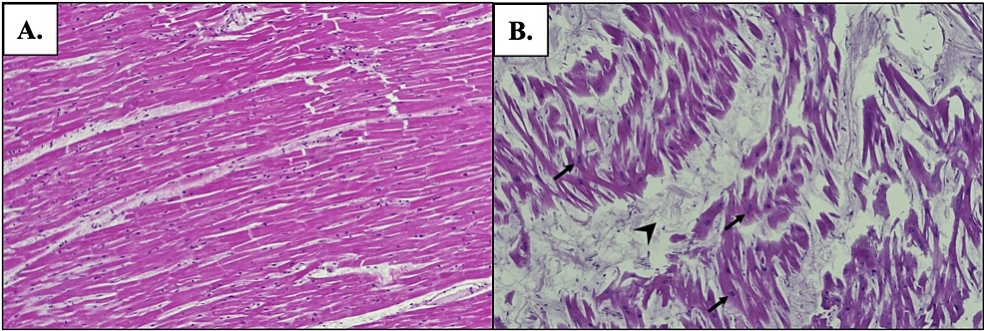
H&E staining of the interventricular septum at 10x magnification A: Unaffected cardiac tissue is characterized by a parallel arrangement of cardiomyocytes; B: Cardiac tissue affected by HOCM shows characteristic myocyte disarray, myofiber hypertrophy, enlarged nuclei (arrows), and abnormal connective tissue within the myocardium (arrowhead) H&E: Hematoxylin and eosin, HOCM: Hypertrophic obstructive cardiomyopathy

**Figure 5:**
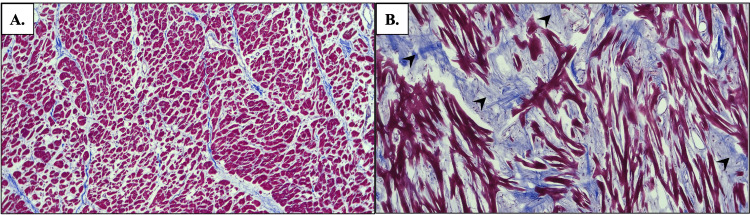
Masson’s trichrome staining of the interventricular septum at 10x magnification A: Unaffected cardiac tissue is without significant accumulation of fibrotic tissue; B: Cardiac tissue affected by HOCM shows myocyte disarray and significant interstitial fibrosis (arrowheads) HOCM: Hypertrophic obstructive cardiomyopathy

Histologic staining of the cardiac tissue further supported the donor’s HOCM diagnosis. The H&E staining was utilized to glean information about the pattern and structure of myocytes. In healthy cardiac tissue, a parallel arrangement of cardiomyocytes with centrally located nuclei is expected (Figure 4A). However, H&E staining of the affected cardiac tissue revealed myocyte disarray, enlarged nuclei, and abnormal interstitial connective tissue within the IVS (Figure 4B). These findings are characteristic of HOCM. Masson’s trichrome staining distinguishes interstitial connective tissue from muscle. Figure 5A displays normal cardiac tissue stained with Masson’s trichrome, whereas staining with Masson’s trichrome of the affected cardiac tissue highlighted significant fibrosis (represented by blue-staining tissue in Figure 5B).

**Figure 6:**
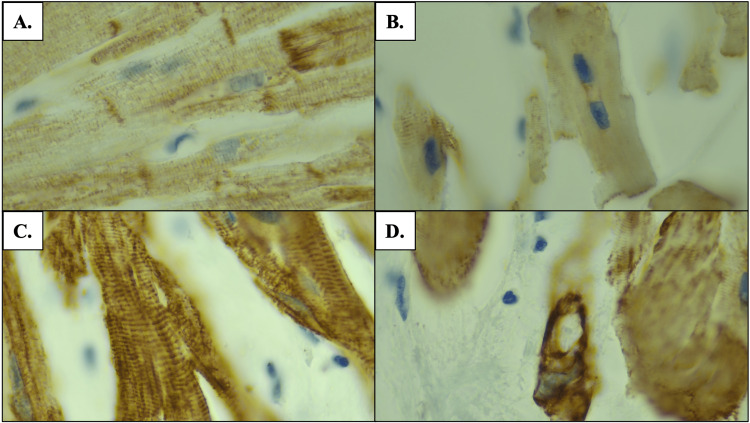
Desmin staining of the interventricular septum at 1000x magnification A: Unaffected cardiac tissue is without histological abnormalities; B: Cardiac tissue affected by HOCM shows decreased desmin staining; C: Cardiac tissue affected by HOCM shows intense desmin staining with positive Z bands; D: Cardiac tissue affected by HOCM shows focal areas of clumping of the desmin filaments HOCM: Hypertrophic obstructive cardiomyopathy

Desmin is an intermediate filament found within cardiomyocytes that helps maintain cell structure and function by linking Z bands to the nuclear membrane, to organelles, and to other Z bands [10]. It is normally distributed in a network around Z bands, intercalated disks, and myofibrils (Figure 6A) [10]. Immunohistochemical staining using antibodies to desmin shows the filament distribution pattern, which is altered in HOCM. Variable patterns of desmin staining have been observed in hearts affected by HOCM, including: (1) decreased or absent staining of Z bands and intercalated discs, (2) areas of intense staining of desmin filaments within myocytes, and (3) areas of parallel arrangement of desmin filaments [10,11]. The findings herein are consistent with those described by D’amati et al. and Francalanci et al. [10,11]. Desmin staining of the interventricular septum of the affected cardiac tissue showed focal areas of decreased staining (Figure 6B), intense staining with visible Z bands (Figure 6C), and clumping of desmin filaments (Figure 6D).

**Figure 7:**
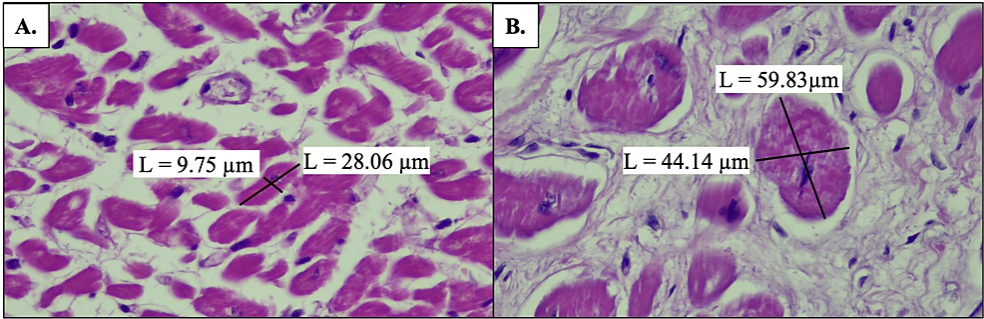
H&E staining of the interventricular septum at 40x magnification A: Micron bars demonstrate normal diameters of a cardiomyocyte in unaffected cardiac tissue; B: Micron bars demonstrate enlarged diameters of a cardiomyocyte in cardiac tissue affected by HOCM H&E: Hematoxylin and eosin, HOCM: Hypertrophic obstructive cardiomyopathy

Lastly, transverse myocyte diameter was found to be larger than is typical (15 µm) in the affected cardiac tissue, measuring >40 µm in some myocytes. The transverse size of a myocyte within the unaffected cardiac tissue measured 9.75 µm by 28.06 µm (Figure 7A) compared to a myocyte within the affected cardiac tissue with dimensions measuring 44.14 µm by 59.83 µm (Figure 7B). Of note, variability was observed in myocyte diameter; however, the cardiomyocytes within the affected IVS were consistently larger than those in the unaffected IVS.

## Discussion

Although the diagnostic modality used to confirm this donor’s premortem HOCM diagnosis is unknown, the findings herein are consistent with the condition. Cardiomegaly, in a male adult, is defined as a heart weight greater than 500 g, which is compatible with our findings [9]. Furthermore, the donor’s heart was 52% heavier than the estimated value for a male of similar BMI (510.1 g vs 335.6 g) [12]. Donor heart weight was also higher than the average values reported by other sources, as shown in Table 2. Of note, the donor’s heart weight is based on that of a formalin-fixed specimen. However, this value is comparable to a fresh specimen as the effects of formalin fixation on tissue weight are negligible within three weeks of embalming [9].

**Table 2:** Comparison of the weight of the HOCM-affected donor heart and the weight of an average male heart HOCM: Hypertrophic obstructive cardiomyopathy

	Donor	Vanhaebost et al. [12] (based on BMI, body surface area, and sex)	Gaitskell et al. [13] (average male)	Gupta et al. [14] (average male)	Molina et al. [15] (based on BMI and sex)
Heart weight (g)	510.1	335.6 (range: 250.3-420.8)	380.0	320.0	308.0 (range: 207-380)

Williams et al. conducted a postmortem analysis on two males with HOCM in whom heart weight was found to be 890 g and 980 g, respectively [16]. When converted into a ratio of heart weight to BMI, which is thought to be a strong predictor of heart weight, the ratios were 34.9 (890/25.5) and 23.6 (980/41.6) [16]. The latter ratio is comparable to the present donor’s ratio at 22.5 (510.1/22.7), suggesting that this case is consistent with prior reports.

When examining the external size of the heart, the longitudinal size was found to be below average while the transverse size was found to be above average (Table 3). While unexpected, it is plausible that fibrotic changes within the interventricular septum may have compromised the longitudinal growth of the heart. When examining internal dimensions, the thickness of the right and left ventricular walls were 4 mm and 16 mm, respectively, and are similar to other reports of HOCM [5]. The thickness of the interventricular septum, at 21 mm, is also greater than the average heart and is consistent with reports of the average septal thickness of a heart affected by HOCM [17]. However, IVS thickness in HOCM is typically threefold greater than that of the LVFW, which was not observed here [1].

**Table 3:** Comparison of heart metrics between HOCM-affected heart and an average heart HOCM: Hypertrophic obstructive cardiomyopathy, IVS: Interventricular septum, RVFW: Right ventricular free wall, LVFW: Left ventricular free wall

Measurement	Donor's heart	Gupta et al. [14]	Kitzman et al. [18]	Standring et al. [19]
Longitudinal size (cm)	9.7	11.3	12.0	12.0
Transverse size (cm)	12.0	8.8	10.5	8.0-9.0
IVS thickness (mm)	21.0	-	14.0	-
RVFW thickness (mm)	4.0	-	4.0	-
LVFW thickness (mm)	16.0	-	12.0	-

Concentric thickening of the left ventricular walls, versus the asymmetric thickening that is characteristic of HOCM, is observed in approximately 10% of HOCM cases [1]. While this donor’s IVS thickness was 31.25% greater than that of the LVFW, the observations herein are more consistent with concentric thickening of the left ventricular walls rather than asymmetric thickening. This is a possible sequela of the patient’s comorbid hypertension, which commonly causes concentric thickening of the left ventricular walls. However, these findings are consistent, by a small margin, with Kitzman et al.’s stance that the ratio between IVS and LVFW thickness in HOCM should be greater than 1.3 [18]. Importantly, this serves as a reminder that a finding of concentric thickening of the left ventricular walls on an echocardiogram or cardiac MRI should not rule out a diagnosis of HOCM.

Abnormal cardiac histology also confirmed the donor’s premortem diagnosis of HOCM, revealing myocyte disarray, myocyte enlargement, nuclear atypia (enlargement and hyperchromasia), and significant interstitial fibrosis. All stains taken together paint a full histologic picture of HOCM. Furthermore, apart from the research conducted by D’amati et al. and Francalanci et al. in the late 1990s, there is a scarcity of literature evaluating desmin staining patterns in HOCM [10,11]. It is the researchers’ hope that these findings will fill this gap in the literature.

## Conclusions

This report suggests that gross and histologic examination of cadaveric cardiac tissue is sufficient to confirm a HOCM diagnosis. Furthermore, the findings herein show that concentric left ventricular hypertrophy is a possible manifestation of HOCM and is not reason enough to exclude HOCM from a differential diagnosis. To the researchers’ knowledge, this is the first report evaluating HOCM in a cadaver donated for medical education.
